# QuickStats

**Published:** 2014-05-16

**Authors:** FROM THE NATIONAL CENTER FOR HEALTH STATISTICS

**Figure:**
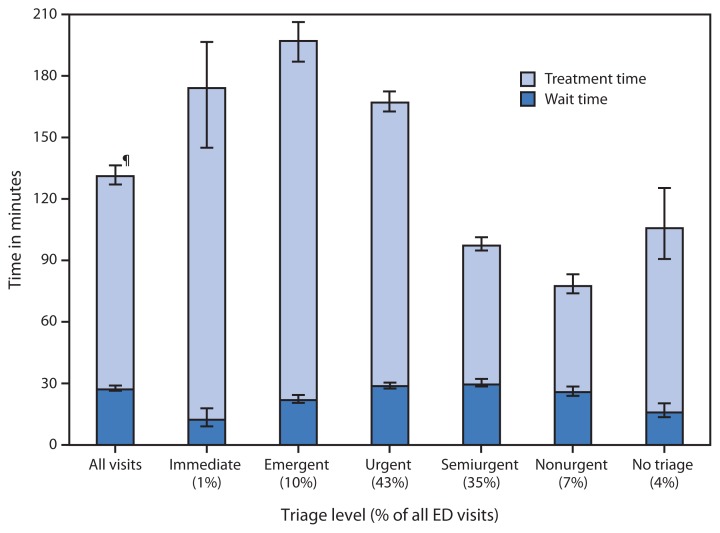
Median Emergency Department (ED) Wait and Treatment Times,* by Triage Level^†^ — National Hospital Ambulatory Medical Care Survey, United States, 2010–2011^§^ * Wait time was defined as the difference between the time of arrival in the ED and the time the patient had initial contact with a physician, physician assistant, or nurse practitioner. Treatment time was defined as the difference between the time the patient had initial contact with a physician, physician assistant, or nurse practitioner and the time the patient was discharged from the ED to another hospital unit or to the patient’s residence. ^†^ Triage level was based on a five-point scale: 1 = immediate, 2 = emergent, 3 = urgent, 4 = semiurgent, and 5 = nonurgent. No triage was defined as a visit to an emergency service area that did not conduct nursing triage. Triage level was imputed for 19.5% of records included in this analysis. Emergency service areas using three or four level triage systems had their responses rescaled to fit the five level system. In 2010 and 2011, rescaling was required for approximately 12.0% of records. ^§^ Estimates are based on 2-year annual averages. Approximately 16.9% of records were excluded from this analysis for the following reasons: patient not seen by a physician, physician assistant, or nurse practitioner; record missing wait or length of visit times; treatment time = 0; or disposition of left after triage, left against medical advice, transferred, or dead on arrival. ^¶^ 95% confidence interval.

The median wait time to be treated in the ED was about 30 minutes, and the median treatment time was slightly more than 90 minutes in 2010–2011. At visits in which patients were triaged, the shortest median wait time was 12 minutes for patients who had an immediate need to be seen. Treatment times were longer for patients who were triaged as immediate, emergent, and urgent compared with those who were triaged as semiurgent or nonurgent.

**Source:** National Hospital Ambulatory Medical Care Survey 2010–2011. Available at http://www.cdc.gov/nchs/ahcd.htm.

**Reported by:** Linda F. McCaig, MPH, lmccaig@cdc.gov, 301-458-4365; Michael Albert, MD.

